# Retraction: Neuropsychiatric Disorders: Bridging the Gap Between Neurology and Psychiatry

**DOI:** 10.7759/cureus.r240

**Published:** 2026-09-01

**Authors:** Sanzida Taslim, Sujeet Shadmani, Abdul Rehman Saleem, Ajay Kumar, FNU Brahma, Narendar Blank, Muhammad Arsalan Bashir, Danya Ansari, Komal Kumari, Muhammad Tanveer, Giustino Varrassi, Satesh Kumar, Arveen Raj

**Affiliations:** 1 Psychiatry, Ross University School of Medicine, Far Rockaway, USA; 2 Medicine, Shaheed Mohtarma Benazir Bhutto Medical College, Karachi, PAK; 3 Psychiatry, Khairpur Medical College, Khairpur, PAK; 4 Internal Medicine, Liaquat University of Medical and Health Sciences, Hyderabad, PAK; 5 Medicine, Indus Hospital and Health Network, Karachi, PAK; 6 Psychiatry, Islamabad Medical and Dental College, Islamabad, PAK; 7 Medicine, New Medical Centre (NMC) Royal Family Medical Centre, Abu Dhabi, ARE; 8 Psychiatry, Lahore Medical and Dental College, Lahore, PAK; 9 Pain Medicine, Paolo Procacci Foundation, Rome, ITA; 10 Psychiatry, Toronto General Hospital, Toronto, CAN

This article has been retracted by the Editor-in-Chief. Following a reader report, a post-publication review found that the article's references do not support its content.

As a review article, its reliability depends on the accuracy of its sources, and its content is therefore unreliable and cannot be corrected. All thirteen authors were invited to respond. One author stated that he did not review or approve the final version prior to publication, the corresponding author could not be reached, and the remaining authors did not respond.

